# Snakes elicit specific neural responses in the human infant brain

**DOI:** 10.1038/s41598-020-63619-y

**Published:** 2020-05-04

**Authors:** J. Bertels, M. Bourguignon, A. de Heering, F. Chetail, X. De Tiège, A. Cleeremans, A. Destrebecqz

**Affiliations:** 1 Consciousness, Cognition and Computation Group (CO3), Center for Research in Cognition and Neurosciences (CRCN), ULB Neuroscience Institute (UNI), Université Libre de Bruxelles (ULB), Brussels, Belgium; 2 Laboratoire de Cartographie Fonctionnelle du Cerveau (LCFC), ULB Neuroscience Institute (UNI), Université Libre de Bruxelles (ULB), Brussels, Belgium; 3 Laboratoire Cognition Langage Développement (LCLD), Center for Research in Cognition and Neurosciences (CRCN), ULB Neuroscience Institute (UNI), Université Libre de Bruxelles (ULB), Brussels, Belgium

**Keywords:** Visual system, Neuroscience, Cognitive neuroscience, Attention, Perception

## Abstract

Detecting predators is essential for survival. Given that snakes are the first of primates’ major predators, natural selection may have fostered efficient snake detection mechanisms to allow for optimal defensive behavior. Here, we provide electrophysiological evidence for a brain-anchored evolved predisposition to rapidly detect snakes in humans, which does not depend on previous exposure or knowledge about snakes. To do so, we recorded scalp electrical brain activity in 7- to 10-month-old infants watching sequences of flickering animal pictures. All animals were presented in their natural background. We showed that glancing at snakes generates specific neural responses in the infant brain, that are higher in amplitude than those generated by frogs or caterpillars, especially in the occipital region of the brain. The temporal dynamics of these neural responses support that infants devote increased attention to snakes than to non-snake stimuli. These results therefore demonstrate that a single fixation at snakes is sufficient to generate a prompt and large selective response in the infant brain. They argue for the existence in humans of an inborn, brain-anchored mechanism to swiftly detect snakes based on their characteristic visual features.

## Introduction

Detecting predators is essential for survival. Over evolution and across species, natural selection may have therefore selected individuals equipped with perceptual systems tuned to detect predators quickly, so enabling better defensive behavior. This hypothesis is at the core of the *Snake Detection Theory*, which posits that the ancient predator-prey relationship between snakes and primates played a substantial role in the evolution and expansion of the latter’s visual system. The vital need to spot snakes rapidly would have shaped primates’ brain such that they developed keen perceptual abilities, and, in particular, the ability to rapidly detect and process visual cues suggestive of snakes^1,2^. The evolutionary pressure exerted by snakes would also have led to the development of a “fear module” in the primate brain — a structure that is selectively sensitive to and automatically activated by evolutionary threat-relevant stimuli, allowing their rapid detection^3^. Evidence for the existence of such a neurobiological substrate for efficient detection of snakes in primates stems essentially from the identification of thalamic neurons in the macaque brain that selectively respond to images of snakes^4^.

Primates would therefore be evolutionarily tuned to swiftly detect and process ancestrally threat-relevant stimuli based on their visual features. Yet, it is unclear whether such detection mechanism is implemented in the naive, immature brain.

Behaviorally, both human and non-human primates are remarkable snake detectors. When presented with an array of pictures, human adults^5–9^ and children^10–15^, but also lab-reared monkeys^16–18^, are faster at detecting a snake among threat-irrelevant pictures than *vice versa*. The coiled body shape of snakes would be a critical feature in attracting participants’ attention^12,14,15^. Remarkably, infants under one year of age also show rapid detection of snake pictures and preferential orienting toward these ancestrally threat-relevant stimuli. When presented with pictures in the visual periphery, infants indeed shift attention faster towards snake pictures than towards threat-irrelevant pictures^19–23^. This visual bias further impacts infants’ processing of subsequent stimuli^19,21^.

Infants’ sympathetic responses to videos, pictures and hissings of snakes likewise support the observation that snakes capture their attention^24–26^. Importantly, these physiological studies, together with other research examining infants’ and toddlers’ approach/avoidance behaviors toward snakes^20,25,27^ reported no fear reaction or signs of distress in these participants. This is clear evidence that fear of snakes is not innate in humans, just as extensive work has previously demonstrated in monkeys (see, e.g.^28^). Primates would rather be *prepared* to learn to fear snakes, so that fear of snakes (and of other evolutionary recurrent threats) is acquired more easily than fear of other non-recurrent threats^29^. Perceptual biases toward snakes – which precede the development of fearful behaviors – would thus act as a catalyst for fear learning^30–32^.

Infant and monkey studies have been taken as strong support in favor of an evolved predisposition to preferentially process snakes. Factually, both infants and lab-reared monkeys are likely fully naive to real snakes, even though infants might have been exposed to stuffed or cartoon versions of such reptiles. Their visual bias towards snakes cannot therefore be attributed to any prior genuine exposure to these animals, and even less to knowledge about their potential dangerousness. It would rather have an evolutionary origin^1^ since primate and human brains would have evolved to detect physical attributes inherent to snakes in priority. These studies are nevertheless scarce and, as regards human studies, have thus far relied exclusively on eye gaze and physiological measures (but see^33^).

In the present study, we used scalp electroencephalography (EEG) to unravel the neuronal mechanisms subtending the evolved predisposition to preferentially and swiftly process snakes. We searched for objective and reliable neural responses to snake images in 7- to 10-month-old infants. To do so, we leveraged fast periodic visual stimulation (FPVS), consisting of trains of four base and one oddball stimuli, thereby tagging oddball frequency at one fifth of base frequency^34,35^. Periodic stimulation is known to generate steady-state visual evoked potentials (SSVEPs) exactly at the same fundamental frequency and harmonics as the driving stimulus^36^. When oddball stimuli are tagged at a fraction of stimulus frequency, the ability of the brain to differentiate between base and oddball stimuli surfaces as periodic responses at oddball frequency. This methodology therefore provides an implicit, objective and predictive measure of stimulus discrimination, and has recently proven its sensitivity in infants exposed to complex visual stimuli^37–39^. Event-related potentials (ERPs) to oddball stimuli were also examined as to uncover the temporal course of the discrimination response. Here, randomly selected color pictures of various animals presented from different viewpoints in their natural background served as base stimuli and were presented at a 6 Hz rate. Oddball stimuli consisting of snake or frog pictures (depending on the type of sequence), equalized for contrast and luminance, were presented every five stimuli (i.e., at 1.2 Hz). Frog pictures were replaced by caterpillar pictures in a control study aiming at testing the specificity of the infant brain response to snakes. Based on previous studies on infants’ visual categorization abilities^40^, we hypothesized that snake-sensitive neural responses would be elicited in 7- to 10-month-old infants, mainly in the occipital region, and would be of larger amplitude than any frog- or caterpillar-sensitive neural responses. The observation of such differential effects would constitute key evidence for the special status of snakes compared to similarly unfamiliar and colorful, but threat-irrelevant animals such as frogs or caterpillars, and would thereby further support the Snake Detection Theory.

## Results

### Snake vs. frog-selective responses in the infant brain

**Figure 1:**
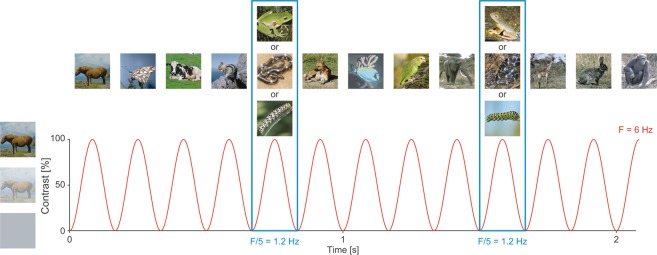
Schematic illustration of the experimental paradigm used. Animal pictures were presented by sinusoidal contrast modulation at a rate of 6 per second (F = 6 Hz). Snake, frog or caterpillar pictures were presented every fifth stimulus (F = 6/5 = 1.2 Hz), in different trial sequences. Snake and frog sequences were used in the main study; snake and caterpillar sequences were used in the control study. The pictures differed in terms of color, viewpoint and lighting conditions. Snake, frog, caterpillar and other animal pictures were equalized in terms of luminance and contrast across the whole set. For copyright reasons, the pictures of snakes, frogs and caterpillars displayed are different than those used in the actual experiment (originally coming from Vanessa LoBue’s personal database), but the degree of variability across images is respected. Most of other animal pictures come from CalPhotos (https://calphotos.berkeley.edu/fauna).

Scalp EEG data were recorded in 26 7- to 9-month-old infants (18 females, mean age = 261 days, SD = 23 days), while viewing 20-s sequences of animal pictures (sinusoidal contrast modulation at a rate F = 6 Hz) (see Fig. 1). An oddball stimulus was presented every fifth stimulus (i.e., F = 6/5 = 1.2 Hz) and consisted in a frog picture in frog sequences, and in a snake picture in snake sequences. Frog and snake sequences were presented in alternation.

Infants viewed between 4 and 20 sequences each (M = 12.19, SD = 3.78). Of the total amount of sequences (n = 317), 88 were excluded on the basis of predetermined criteria, and 11 snake sequences were randomly discarded to ensure comparability of SNR measures between conditions (see Methods). Final analyses were therefore run on 109 snake and 109 frog sequences.

#### Frequency domain analyses

##### Base frequency:

**Figure 2:**
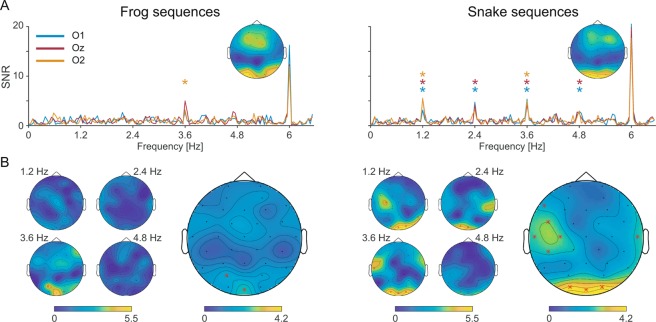
Frequency-domain representation of frog and snake-selective responses during fast periodic visual stimulation (left and right panels, respectively). (**A**) SNR spectra of each occipital electrode (O1, Oz, O2) and topographical maps of SNR at the base frequency (6 Hz). Asterisks indicate significant oddball responses. (**B**) Topographical maps of SNR at each harmonic of the oddball frequency (1.2, 2.4, 3.6, and 4.8 Hz; left part), and of SNR averaged on these first four harmonics (right part). Asterisks indicate significant responses at specific channels.

In accordance with our selection of sequences (see Method), grand-averaged signal to noise (SNR) spectra showed clear responses at the first and second harmonics of animal pictures presentation rate (6 and 12 Hz, respectively). These responses were characterized by a medial occipital topography peaking for both frog and snake sequences at electrode O1 at 6 Hz (mean SNR in the frog sequence = 16.26; mean SNR in the snake sequence = 20.52, see Fig. 2A), and at Oz at 12 Hz (mean SNR in the frog sequence = 11.14; mean SNR in the Snake sequence = 10.87).

Comparisons between SNR values in frog and snake sequences at 6 Hz at channel O1, and at 12 Hz at channel Oz, did not reveal any significant difference (*ps* > 0.10; permutation statistics). These results indicate that infants’ visual system synchronized successfully to the rapid presentation of animal pictures, and that this synchronization did not differ significantly between sequences.

##### Oddball frequency (discrimination response):

Considering the SNR values averaged across the four harmonics before base frequency (i.e., 1.2, 2.4, 3.6 and 4.8 Hz; see Methods for justifications) for frog sequences, analyses revealed a significant response to frog pictures at electrodes PO3 and Oz (mean SNR = 1.84 and 2.27, *ps* < 0.05, see Fig. 2B).

Similar analyses revealed significant responses to snake pictures at occipital (O1, Oz and O2; mean SNRs = 4.03, 4.11 and 4.17, *ps* < 0.005) and fronto-temporal channels (F7, FC5, T7, C3, CP5, and T8; mean SNRs between 1.84 and 2.68, *ps* < 0.05; see Fig. 2B).

Examining the SNR values of each harmonic separately (see Fig. 2A,B), a significant response to frog pictures was found at parieto-occipital sites (PO3 and O2) at the third harmonic (F = 3.6 Hz; mean SNRs = 4.60 and 3.18, respectively, *ps* < 0.05). No other significant response was observed at any electrode at the oddball frequency and harmonics. Significant responses to snake pictures were observed up to the fourth harmonic (i.e., at 1.2, 2.4, 3.6 and 4.8 Hz), at the medial occipital lobe (O1 and Oz, mean SNRs = 2.87–5.36 and 2.50–5.52, respectively; *ps* < 0.05, see Fig. 2A). At 1.2 Hz, significant responses were also recorded at electrodes O2, C3 and P8 (mean SNRs = 5.56, 4.24 and 3.47, respectively, *p*s < 0.05). At 3.6 Hz, a significant response was also observed at electrodes O2 and T7 (mean SNR = 5.01 and 4.15, *p* < 0.05).

To quantify differences between frogs and snakes category-specific responses, we contrasted SNR values averaged across the first four oddball harmonics in frog and snake sequences. Significant differences emerged between averaged SNR values in frog and snake sequences at electrodes O1, Oz, O2 and CP5 (*p*s < 0.03).

#### Time-domain analyses

We then explored EEG signals in the temporal domain to characterize the spatio-temporal dynamics of selective responses to frogs and snakes.

The general response pattern for both frog and snake pictures was reminiscent of the typical pattern induced by visual stimulation in infants, namely a negative deflection at around 200–300 ms (i.e., the N290), followed by a sustained positivity at around 400–600 ms (i.e., the P400), recorded over medial occipital regions^41^. Nevertheless, after the appearance of frog pictures, only the time-window at around 450–500 ms proved to be significant at O1 (*p* < 0.05) and peaked at 470 ms. Similarly, after the appearance of snake pictures, only the time-window at around 390–570 ms proved to be significant (*p*s < 0.05 for O1, Oz and O2, but also for FC5, C3, CP5, P8 and C4). This positive component peaked at 460 ms over occipital channels.

**Figure 3:**
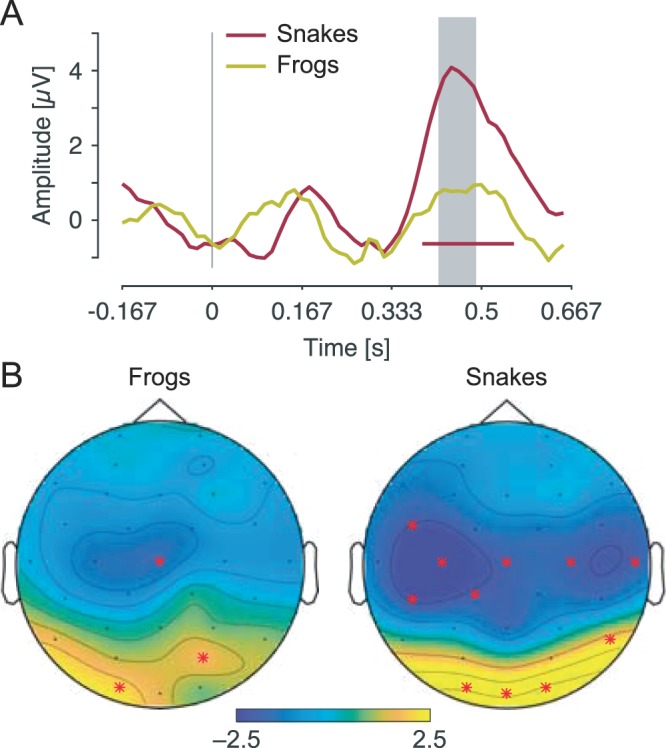
Time-domain representation of frog and snake-selective responses during fast periodic visual stimulation. (**A**) Grand averages of the notch-filtered EEG responses relative to the onset of the frog and snake stimuli, at O2. The red line below the waveforms represents the time-points at which the signal significantly deviates from baseline after snake pictures. The grey area indicates the time-window at which the signal significantly differs between frog and snake pictures. Note that the amplitude scale is only approximate due to the epoch-normalization scheme used (see methods). (**B**) Topographical maps of the P400 evoked by frog and snake stimuli in the time-window at which the signal significantly differs between them (i.e., 420–490 post-stimulus onset).

Importantly, the amplitude of this component was significantly larger at O2 when a snake than when a frog picture was presented (*p* = 0.01, at around 420–490 ms). Figure 3 displays the notch-filtered responses at O2 for both frog and snake pictures.

Of note, the shape of the temporal response to frogs (i.e., 3 peaks and troughs of roughly similar amplitude) explains why the third harmonic was predominant in SNR spectra.

### Replication and clarification of the snake-specific visual features driving the effect

The aim of this control study was two-fold. First, we wanted to ensure that the observed snake-specific infant brain response was robust and not due to a novelty or pop-out effect of snakes (all having similar elongated limbless coiled body shapes). Indeed, most of other presented animals, compared to snakes and despite their high variability, were limbed (77% were quadrupeds) and had rather collected body shapes. Second, we wanted to specify the critical features of snakes driving the specific brain response. In particular, we wanted to test the possibility that their prototypical curvilinear coiled shape is important, as has been evidenced in previous behavioral studies^12,14,15^. To do so, we exposed another smaller group of 7- to 10-month-old infants (n = 13, 8 females, mean age = 272 days, SD = 43 days) to alternating snake and caterpillar sequences (i.e., in non-snake sequences, frog pictures from our main study were replaced by caterpillar pictures, see Fig. 1). Caterpillars were chosen as control stimuli since, as snakes, they have long bodies and no prominent legs^15^. They thereby visually differ from the animals used as base stimuli, as snakes do. Crucially, the coiled posture is not characteristic of caterpillars; therefore none of our pictures depicted coiled caterpillars. Hence, if the effect observed in the main study for snakes depends on specific features of these animals such as their coiled shape – as previous studies have suggested and as predicted by the Snake Detection Theory, one might expect to replicate the effect observed in our main study, namely an increased response to snakes than to non-snake animals (here, caterpillars). On the contrary, if the infant brain response to snakes observed in the main study is due to their elongated shape (and thereby reflects an effect of unspecific features of snakes) or to the contrast between that shape and the collected shapes of the other animal pictures, similar effects should be observed for snake and caterpillar pictures in the present experiment.

Infants viewed between 9 and 23 sequences each (M = 12.92, SD = 3.68). Of the total amount of sequences (n = 168), 57 were excluded on the basis of fixed criteria, and one caterpillar sequence was randomly discarded to ensure comparability of SNR measures between conditions (see Methods). Final analyses were therefore run on 55 snake and 55 caterpillar sequences.

Given the smaller sample of this control study compared to the main study, SNRs were obviously lower. We therefore used *a priori* hypotheses based on results from the first study. That is, for the appraisal of responses in the frequency and time domains, we assessed only the responses at occipital electrodes, further averaged across the four harmonics for the frequency domain.

#### Frequency domain analyses

##### Base frequency:

**Figure 4:**
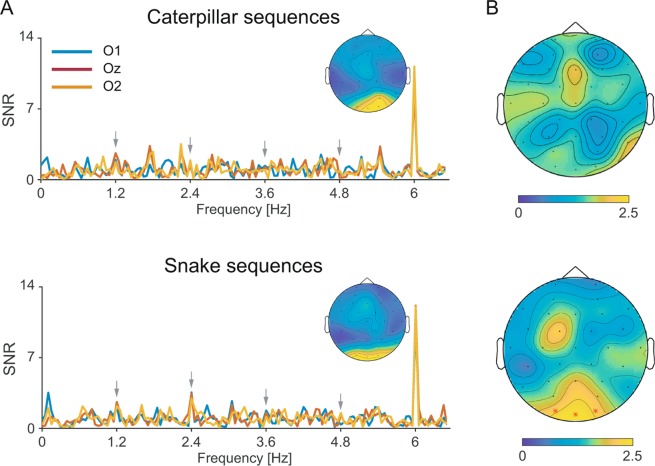
Frequency-domain representation of caterpillar and snake-selective responses during fast periodic visual stimulation (left and right panels, respectively). (**A**) SNR spectra of each occipital electrode (O1, Oz, O2) and topographical maps of SNR at the base frequency (6 Hz). (**B**) Topographical maps of SNR averaged on the first four harmonics.

In accordance with our selection of sequences (see Method), grand-averaged SNR spectra showed clear responses at the first and second harmonics of animal pictures presentation rate (6 and 12 Hz, respectively). These responses were characterized by a medial occipital topography peaking at 6 Hz at electrode O2 for caterpillar sequences (mean SNR = 11.19) and at electrode Oz for snake sequences (mean SNR = 12.14), and at 12 Hz at electrode Oz for both caterpillar and snake sequences (mean SNR in the caterpillar sequences = 10.43, mean SNR in the snake sequences = 14.7, see Fig. 4A).

Comparisons between SNR values in caterpillar and snake conditions at 6 Hz at channel O2 and Oz, and at 12 Hz at channel Oz, did not reveal any significant difference (*ps* > 0.50; permutation statistics).

##### Oddball frequency (discrimination response):

Considering the SNR values averaged across the four harmonics before base frequency (i.e., 1.2, 2.4, 3.6 and 4.8 Hz) for caterpillar sequences, analyses did not reveal any significant response to caterpillar pictures (all *ps* > 0.30, see Fig. 4B). Similar analyses revealed significant responses to snake pictures at all three occipital channels (mean SNR at O1 = 1.97, mean SNR at Oz = 2.24 and mean SNR at O2 = 1.95, *ps* < 0.05, see Fig. 4B). Of note, when taking into account the complete set of electrodes as in Experiment 1, analyses revealed very similar results: no significant response to caterpillar pictures (all *p* > 0.10), but significant responses to snake pictures at Oz and O2 (*ps* < 0.05).

When contrasting SNR values averaged across the first four oddball harmonics in caterpillar and snake sequences at occipital electrodes, a significant difference emerged between averaged SNR values in caterpillar and snake sequences at electrode Oz (*p*s < 0.05). The difference at electrodes O1 and O2 did not reach significance (*p* > 0.05). This analysis further supports the existence of snake-selective brain responses that are higher in amplitude than (non-significant) caterpillar responses.

#### Time-domain analyses

**Figure 5:**
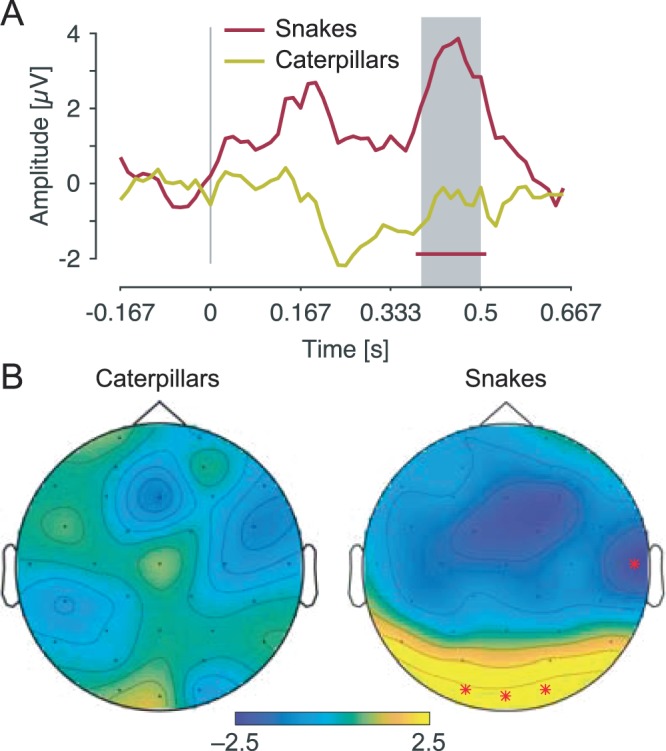
Time-domain representation of caterpillar and snake-selective responses during fast periodic visual stimulation. (**A**) Grand averages of the notch-filtered EEG responses relative to the onset of the caterpillar and snake stimuli, at O2. The red line below the waveforms represents the time-points at which the signal significantly deviates from baseline after snake pictures. The grey area indicates the time-window at which the signal significantly differs between caterpillar and snake pictures. Note that the amplitude scale is only approximate due to the epoch-normalization scheme used (see methods). (**B**) Topographical maps of the P400 evoked by caterpillar and snake stimuli in the time-window at which the signal significantly differs between both (i.e., 390–500 ms post-stimulus onset).

As in the first study, although the general response pattern for snake pictures was reminiscent of the typical pattern induced by visual stimulation in infants, only the time-window at around 380–510 ms after the appearance of snake pictures proved to be significant (*p*s < = 0.001 for O1, Oz and O2). This positive component peaked at 445 ms over occipital channels. The appearance of caterpillar pictures did not evoke significant EEG responses (all *p* > 0.25). Importantly, the amplitude of the response in the occipital region was significantly larger at O2 when a snake than when a caterpillar picture was presented (*p* = 0.004, at around 390–500 ms). The amplitude difference at O1 and Oz did not reach significance (*p*s = 0.09). Figure 5 displays the notch-filtered responses at O2 for both caterpillar and snake pictures.

## Discussion

Recent behavioral and psychophysiological studies have shown that human infants, like their older peers, are remarkable snake detectors^19–23^. Given infants’ innocence regarding the threat that snakes represent, these findings support the idea that humans have a phylogenetic predisposition to rapidly detect evolutionarily threat-relevant stimuli based on their physical attributes, originating from our ancestors’ vital need to spot predatory snakes rapidly^1^. The present study provides novel electrophysiological evidence that this predisposition is subtended by a neurobiological substrate that is functional early in development, independent of any prior experience with snakes, and sensitive to snake prototypical features.

Using a fast periodic visual stimulation approach, we examined infants’ neural responses to periodic oddball pictures of snakes and non-snake control animals. Frogs were used as controls in the main study as they are similarly unfamiliar, colorful and shiny, but threat-irrelevant compared to snakes^15^. Both types of stimuli generated a periodic response in the posterior region of the infant’s brain. Critically, glancing at snakes automatically generated neural responses that were higher in amplitude and more widespread than those generated by frogs. This differential effect was replicated in a control study where we contrasted infant brain responses to snakes and caterpillars, using the same design with a different smaller group of participants. These findings strongly support the notion that humans have an early propensity – a possibly inborn predisposition – to rapidly detect specific visual features of snakes.

Higher response to a specific stimulus category has been previously related to its relevance compared to other visual categories^42^. Here, in line with the idea that the ancient predator-prey relationship between snakes and primates shaped our visual system so that they are detected rapidly^2,3,43^, we argue that the evolutionary threat-relevance of snakes compared to frogs and caterpillars is responsible for the enhanced response to snakes. The fact that stronger neural activity was observed precisely in occipital areas strongly supports the idea that what underlies this snake-selective discrimination response mainly relates to their visual physical attributes, echoing results from behavioral studies^14,15,23,44,45^. Since infants are innocent regarding the snake matter, and given that previous studies have clearly demonstrated that fear of snakes is not innate^20,25,27^, infants’ brain responses when seeing a snake would not be linked to any fear or threat detection but rather to the detection of physical features inherent to snakes that the primate and human brain would have developed to detect quickly and in priority. It has been proposed that evolution shaped humans to develop visual templates for rapid recognition of animals that represent a threat for their survival^46^. These templates would integrate the low-level features and shapes of animals that have been associated with danger throughout evolution, enabling their rapid identification.

The elongated coiled shape of snakes (common to all snakes in our study and characteristic of snakes in their natural state) is most probably a distinctive and instrumental visual feature of these animals^14,44^. When responses to coiled snakes were contrasted with responses to caterpillars – that also have elongated but not coiled body shapes, snake-specific but not caterpillar-specific responses were observed in the occipital area. The absence of responses to caterpillar pictures could be attributable to a lack of power since we had two times less sequences for caterpillars than for frogs. In any case, responses to snakes differed from (non-significant) responses to caterpillars. These results therefore evidence that infant brain responses to snakes are robust, and specific to snakes. Importantly, they also demonstrate that snake-specific responses are not due to a novelty or “pop-out” effect of their elongated body shape when presented among animals that, for the vast majority, have prominent limbs and a collected shape. Otherwise, similar responses to caterpillar pictures would have also been observed.

Considering the high presentation rate and the heterogeneity (i.e., in terms of color, background and winding style) of the snakes presented in this study, detection of snake prototypical attributes appears to be a highly efficient process. A possibility is that the infant brain actually responds to a combination of physical traits, or to even more low-level features. For instance, recent studies have shown that snake scale patterns also are a determinant factor in the fast detection of snakes by human and non-human primates^47–49^. In the pictures used in the present study, snake scales are visible, although not close-up. At a more basic level, a core spectral feature of snakes that is related to their coiled body shape – i.e., their high contrast energy in midrange spatial frequencies – has been recently spotted as likely responsible for their conspicuity^49,50^. Interestingly, this feature is shared by many poisonous animals, which supports the adaptive value of a fast-acting visual mechanism responding to it^50^. Nevertheless, as a counterpart, any stimulus that coincidently possesses this core spectral feature (although not being inherently threat-relevant, such as holes^50^ or coiled wires^14^) might induce some form of aversion or fast detection, just as snakes do, because of the survival value of such behavior^50^. In this view, the infant brain should also respond to non-snake stimuli depicting prototypical snake features. Still, the fact remains that the infant brain reacts strongly to snake-like features, not to frog- or caterpillar-like features, and this effect would most probably have evolutionary grounds.

Although differing in their amplitude, both snake and frog pictures generated a periodic response in the posterior region of the infant brain, associated to a sustained positive ERP component. These results provide further evidence that 7- to 9-month-old infants can categorize stimuli at a basic level^40^ (in this case, animals according to species). A single fixation at these animals (i.e., pictures remained on screen less than 200 ms) was indeed sufficient for the infants’ brain to discriminate these species from others, and to generalize across different exemplars of the same species. However, caterpillar pictures did not evoke any significant response. Although this null result might be due to a lack of power (we had half of the sequences in Exp. 2 than in Exp. 1), it could also indicate that the ability to categorize animals by species develops with age, with some species categorized more readily than others. Future studies should examine this possibility further.

Examining the temporal dynamics of the neural responses revealed that snake pictures evoked a stronger P400 component than frog and caterpillar pictures. The enhancement of an ERP component can generally be interpreted as reflecting an attentional effect^51^. This suggests that increased attention is devoted to snakes compared to frogs or caterpillars, though at a rather late stage of neocortical processing. Crucially, it provides novel electrophysiological correlates for prior behavioral findings demonstrating that snakes capture infants’ attention^19,21,22^. In fact, as suggested elsewhere^52^, the higher P400 could reflect feedback from anterior attentional networks to posterior perceptual systems. Source analyses of the P400 component have indeed suggested that anterior brain regions associated with attention contribute to a sustained P400 response in infants^53^. Periodic responses to snakes recorded in fronto-temporal regions in the first study could precisely reflect the activation of these anterior sustained attention networks.

The increase in P400 amplitude in response to snakes is also in line with previous findings of a higher P400 response elicited by fearful than neutral and happy faces in 7-month-old infants^54^. Although faces differ from snakes by their social nature and their high familiarity to infants, both are of great adaptive relevance for our species, and preferential detection of face-like patterns has been shown to be independent from experience^55,56^. Moreover, fearful faces, just as snakes, are evolutionarily threat-relevant stimuli. In this respect, both might preferentially engage the subcortical visual system (involving the superior colliculus and the pulvinar for fast access to the amygdala) that would be responsible for the rapid detection of and fast response to ancestral survival threats^43^; although this view is still debated (see^57^ for further discussions). One possibility is that the observed snake-sensitive increase in P400 amplitude would stem from the fast involvement of the subcortical visual system, which would in turn trigger subsequent attentional biases towards snakes at the occipital level through extensive connectivity with visual and associative neocortical sites^57^.

Admittedly, although we can be sure that none of our participants had previous experience with living snakes nor were particularly familiar with these species (as confirmed by their parents), we cannot rule out the possibility that, despite their young age, they have been exposed to representations of snakes, such as in picture books or through stuffed toys. However, it is very unlikely that, at that age, the caregiver pointed the potential dangerousness of the animal depicted. Moreover, there is no reason to believe that such innocuous exposition to animal representations would have occur more for snakes than for frogs or caterpillars. Replicating our findings in newborns would nevertheless provide unequivocal evidence that brain responses to snakes are inborn, and functional from birth.

Overall, this study argues for the existence in humans of an inborn brain-anchored mechanism to swiftly detect snakes, based on their perceptual features. It provides novel electrophysiological evidence supporting the Snake Detection Theory by demonstrating that neural systems enabling humans to detect evolutionarily relevant threats are functional early in life, independent of any prior experience and sensitive to snake prototypical features. More broadly, together with recent evidence that inexperienced chicks use visual cues to adequately respond to different threats^58^, these results support the notion that the visual system of different species might have been shaped by the need to detect predators and develop appropriate reactions.

## Materials and methods

### Experiment 1

#### Participants

Twenty-six full term 7- to 9-month-old infants (18 females) composed the final sample (mean age = 261 days, SD = 23 days). Five additional infants were tested but excluded from the analysis (one due to excessive crying and four because their data indicated that they did not attend any of the sequences in at least one condition, see below). The infants selected neither exhibited known developmental difficulties nor were they particularly familiar with frogs or snakes, as revealed by a questionnaire completed by the accompanying parent (though we cannot exclude that they have been exposed to representations of frogs or snakes, such as in picture books or through stuffed toys). The parents gave informed consent prior to testing. The Psychological and Educational Sciences Faculty Ethics Committee of the Université libre de Bruxelles, and the CUB Hôpital Erasme Ethics Committee approved the experimental protocol. The methods were carried out in accordance with the approved guidelines and regulations.

#### Stimuli and procedure

Figure 1 illustrates the experimental design. Infants were presented with visual stimuli consisting of colored pictures of animals presented in their natural background, from different viewpoints. Base stimuli consisted of 130 animal pictures from 13 different species (dogs, cats, fishes, rabbits, horses, monkeys, squirrels, cows, birds, gazelles, elephants, giraffes and butterflies; 10 exemplars of each) collected from the internet (mostly from https://calphotos.berkeley.edu/fauna). Oddball stimuli consisted of 29 snake and 29 frog pictures used in a previous study^15^. Snakes were all depicted coiled to maximize the animal/background ratio. None of them was in a manifest attack posture. Frogs have often been used as control non-snake animal stimuli^15^. They indeed resemble snakes in texture, brightness and color, and can be considered as similarly unfamiliar for infants as snakes are. Pictures were resized to 200 × 200 pixels, and equalized in terms of luminance and contrast across the whole set using Matlab (Mathworks, USA) to minimize low-level features.

Pictures were displayed at the center of a 60 Hz and 800 × 600 pixel resolution monitor, on a light grey background. At a looking distance of 40 cm, they subtended approximately 13 × 13 degrees of visual angle.

Stimuli were presented at a rate of 6 Hz (base stimulation frequency) using the Psychtoolbox for Windows in Matlab 2013b (MathWorks Inc.). The stimulation cycle of each picture therefore lasted 166.7 ms (i.e., 1000 ms/6) and began with a uniform grey background. Stimuli were presented through sinusoidal contrast modulation (0–100%). Full contrast therefore reached its maximum at 83.35 ms (see Fig. 1).

Stimuli were presented in sequences lasting 20.83 s, which were flanked by a 1.67-s fade-in at the beginning of the sequence, and by a 1.67-s fade-out at its end. This resulted in a total of 145 pictures per sequence, all of which were different within sequences.

Each sequence consisted of successions of series of 4 base animal pictures and 1 oddball stimulus always presented right after. Oddball stimuli were either frog pictures for frog sequences, or snake pictures for snake sequences. Pictures were randomly selected from each set. Frog and snake sequences were presented in alternation. The first sequence was chosen randomly. As a result, 16 in 26 infants started with a frog sequence. This manipulation created a trial sequence containing changes at a frequency of 1.2 Hz (6 Hz/5) that could be directly identified in the EEG spectrum as an index of discrimination by the infant’s visual system of frogs or snakes.

Infants were seated in a car seat in a dimly lit and quiet room. Parents were seated behind them and instructed not to interact with their child. Infants viewed as many sequences as they were inclined to (M = 12.19, SD = 3.78, range = 4–20). Looking behavior was monitored during the experiment by means of a webcam attached to the computer screen. The experimenter initiated each sequence manually once the infant started looking at the screen. When the infant looked away from the screen, the experimenter attracted his/her attention by means of her voice or of a bell. If needed, breaks were provided between sequences. Testing took between 2 and 10 minutes overall.

#### EEG acquisition

EEG signals were acquired at 1024 Hz using a BioSemi system (Amsterdam, Netherlands) with 32 electrodes arranged according to the standard 10–20 system locations and two additional reference electrodes. Electrode offset was reduced to between ± 25 *μ*V for each individual electrode by injecting the electrode with saline gel.

Triggers were sent at the start of each sequence, indicating the type of sequence the participant was exposed to (i.e. frog or snake sequence), and in between all successive images (i.e., when contrast is 0%).

#### EEG pre-processing

EEG pre-processing was carried out using Letswave 5 (http://letswave.org) running on MATLAB R2017a (The Mathworks), following previously described procedures (see, e.g.^34,38^).

EEG data were first filtered through 0.1–100 Hz using a FFT band-pass filter. Filtered data were then downsampled to 250 Hz to reduce data processing time. Trials were extracted from 2 s before sequence onset to 2 s after sequence offset (which served as baseline, see, e.g.^38^), resulting in 28.17 s segments. Sequences were further examined in the time domain for possible channel artifacts. Noisy channels were reconstructed by linear interpolation of surrounding channels (for a maximum of three channels per infant). Electrode interpolation was applied in 15 infants. A common average reference computation was then applied to all channels. Pre-processed data segments were then trimmed to exclude the fade-in and fade-out periods, resulting in 20.83-s stimulation sequences (25 cycles, 5210 bins in total).

A Fast Fourier Transform (FFT) was then applied to these segments. Frequency resolution (i.e., the interval between adjacent frequency bins) was of 1/20.83 s = 0.048 Hz. For each sequence, signal-to-noise (SNR) responses were computed as the ratio between the amplitude at each frequency bin and the average amplitude at the 12 surrounding frequency bins (6 on each side, excluding the immediately adjacent bins, see^37,39^). SNR values significantly above 1 at oddball frequency would indicate specific discrimination of the oddball stimuli.

EEG sequences were discarded when the infant did not look at the screen for the majority of the sequence (as noted online by the experimenter and further supported by the video recording). Only sequences with an SNR above 2 at the base frequency in at least one of the medial occipital electrodes (O1, O2, Oz) were kept for analyses (see^37^ and^38^ for similar procedures), the rationale being that the infant brain would not synchronize to the stimulation frequency if (s)he is not looking at the screen. On average, ~3 sequences were excluded per infant (M = 3.38, SD = 2.64, range 0–9). There was no statistical difference in the number of sequences kept in the frog (M = 4.19, SD = 2.14, range = 1–8) and snake conditions (M = 4.62, SD = 1.86, range = 2–8; *p* > 0.10). We were left with a total of 120 snake sequences and 109 frog sequences across all infants. Of the snake sequences, 11 were randomly discarded to ensure comparability of SNR measures between conditions.

#### Frequency domain analyses

Further data processing was carried out with custom-made MATLAB code. A Fast Fourier Transform (FFT) was applied to each of the kept sequences (109 snake and 109 frog sequences). For each sequence, Fourier coefficients were normalized by their mean amplitude across 0.6–1.8 Hz, a frequency range that surrounds oddball frequency (~1.2 Hz). This procedure minimized the impact of epochs contaminated by excessive movement artifacts by giving about the same weight to all sequences at ~1.2 Hz. For each condition and electrode, amplitude spectra were obtained as the modulus of the averaged Fourier-transformed sequences. Averaging was performed both across all sequences of a given type (frog or snake), yielding group-level amplitude spectra, and within subjects, yielding subject-level amplitude spectra. Note that because the modulus was taken after averaging Fourier coefficients, our derivation of amplitude spectra allowed for phase cancellation of activity not phase-locked sequences. SNR responses were derived from the amplitude spectra as described above.

For the sake of statistical analysis, Z-scores were also calculated as the difference between amplitude (group-level or subject-level) at each frequency bin and mean amplitude at the 12 surrounding frequency bins (excluding the immediately adjacent bins, see below) divided by the standard deviation of the amplitude at these 12 surrounding bins. We hypothesized that snake pictures would generate stronger responses than frog pictures at oddball frequency and harmonics.

#### Time-domain analyses

Periodic snake and frog responses were also investigated in the time domain, even though the rapid presentation rate of the stimuli (resulting in overlapping evoked responses) and their diversity do not provide ideal conditions for the investigation of event-related responses.

Preprocessed 20.83-s sequences were band-pass filtered through 0.5–29 Hz (zero phase shift Butterworth filter, order 4), and notch filtered to selectively remove the contribution of the base stimulation frequency and its first four harmonics (6 to 24 Hz, FFT filter with a Hanning window of 0.1 Hz width, see^42^ for a similar procedure). Sequences were then segmented into 24 epochs of exactly one cycle duration (5 pictures). Epochs started 166.7 ms before the onset of the category-specific event (−166.7 to 666.8 ms). Epochs were normalized by their root-mean-square amplitude and averaged for each condition separately. Baseline correction was applied by subtracting the mean amplitude in the −166.7 to 0 ms time-window.

We expected to observe larger amplitudes for snakes than frogs in the typical deflections induced by visual stimuli in the second half of the first year of life, namely a negative deflection at around 200–300 ms (i.e., the N290), followed by a sustained positivity at around 400–600 ms (i.e., the P400), at occipito-temporal sites^41^.

#### Statistics

A non-parametric permutation-like test was used to estimate the statistical significance of response amplitude (group-level or subject-level) at oddball frequencies^59^. It specifically tested the null hypothesis that oddball stimuli elicit similar response as base stimuli, for each type of sequence separately. The test sought for significant response at all electrodes, with correction for multiple comparisons across electrodes. Such statistical test was chosen because it can support claims of statistically significant effect at each electrode separately, in contrast with, e.g., cluster-based permutation tests^60^. Given that we did not expect multiple harmonics to have a direct meaning in terms of underlying pathophysiological processes^61,62^, the response considered was the average of the responses across the four first harmonics of oddball frequency (1.2, 2.4, 3.6, and 4.8 Hz) in a first step, and the response at each of these individual frequency bins in a second step. Practically, the mean Z-scores across tested frequencies (one value per electrode) were computed based on sequences in which either the first or last cycle was removed. A permutation distribution was then built by estimating 1000 times the maximum – across tested electrodes – of the mean Z-score across tested frequency bins derived from sequences randomly trimmed by a duration corresponding to the *n* = 0, 1, 2, 3, 4 first images (*n* × 1/(1.2 Hz)) and 5–*n* last images. Trimming the sequences randomized the position of the oddball images while preserving synchrony in image presentation across sequences. Hence, this procedure destroys the phase locking – across sequences – of possible responses specific to oddball images, that is needed for peaks at oddball frequencies to show in amplitude spectra. The significance of the response at each tested electrode was computed as the proportion of values in the permutation distribution that were above the observed value. This test, being akin to a permutation test^59^, is exact, and because the permutation distribution was built on maximum values across electrodes, it intrinsically deals with the multiple comparison issue.

A similar test was used to compare group-level responses between both types of sequences at oddball and base frequencies separately. In that test, the Z-scores (oddball frequencies, mean across 1.2, 2.4, 3.6 and 4.8 Hz and each of these frequencies in isolation; base frequency, 6 Hz and 12 Hz separately) – derived from untrimmed sequences – were contrasted between conditions, and this contrast was compared to a permutation distribution in which the maximum across all electrodes of such contrast value was obtained for 1000 random shuffles of frog and snake sequences.

Permutation tests similar to those used in the frequency domain were used to assess the significance of group-level responses in the time domain. In these tests, responses in all electrodes were compared to a permutation distribution in which the maximum across tested electrodes and all time points (from –167 to 667 ms) of such response was obtained for 1000 random trimmings of the sequences.

Permutation tests were also used to assess the significance of the contrast between conditions in the time domain. To increase statistical power, the statistical assessment was performed on the mean of the contrast response across the occipital electrodes. This response was compared to a permutation distribution in which the maximum across time points of such response was obtained for 1000 random shuffles of frog and snake sequences.

### Experiment 2

#### Participants

Thirteen full term 7- to 10-month-old infants (8 females) composed the final sample (mean age = 272 days, SD = 43 days). Three additional infants were tested but excluded from the analysis (one because his·her data indicated that he·she did not attend any of the sequences in at least one condition, and two because more than three electrodes had to be interpolated). The infants selected neither exhibited known developmental difficulties nor were they particularly familiar with caterpillars or snakes. Prior to testing, written informed consent was obtained from the parent for involving his·her child in the study and completing the questionnaire. Same Ethical Committees as in Experiment 1 approved the experimental protocol.

#### Overall procedure and analyses

Most of the procedure and analyses was identical to that used in Experiment 1. Below, we list the differences.

Frog pictures were replaced by caterpillar pictures. Stimuli were presented in sequences lasting 20 s, which were flanked by a 2-s fade-in at the beginning of the sequence, and by a 2-s fade-out at its end. Caterpillars were chosen as non-snake control stimuli since, as snakes, they have long bodies and no prominent legs, but cannot coil themselves^15^.

Infants viewed an average of 12.92 sequences (SD = 3.68, range 9–23). Testing took between 4 and 10 minutes overall.

In EEG pre-processing, we extracted 20-s stimulation sequences that comprised 24 cycles of five pictures (4 base and 1 oddball). The frequency resolution was of 1/20 s = 0.05 Hz. On average, ~4 sequences were excluded per infant (M = 4.38, SD = 2.75, range 1–8). There was no statistical difference in the number of sequences kept in the caterpillar (M = 4.31, SD = 2.06) and snake conditions (M = 4.23, SD = 2.05; *p* > 0.10). We were left with a total of 55 snake sequences and 56 caterpillar sequences across all infants. One caterpillar sequence was randomly discarded to ensure that SNR measures are comparable between conditions.

The datasets generated during and/or analyzed during the current study are available from the corresponding author.
